# Comparison of stereotactic body radiation therapy versus fractionated radiation therapy for primary liver cancer with portal vein tumor thrombus

**DOI:** 10.1186/s13014-021-01874-7

**Published:** 2021-08-14

**Authors:** Sujing Zhang, Li He, Changwen Bo, Shufang Yang, Yonghui An, Na Li, Yingchun Zhao, Liping Zhao, Wenhua Ma, Zheng Zheng

**Affiliations:** 1 Department of Oncology, The First Hospital of Hebei Medical University, No.89 Donggang Road, Yuhua District, Shijiazhuang, 050031 Hebei China; 2 Department of Laboratory Medicine, Handan Hospital of Traditional Chinese Medicine, Handan, Hebei China

**Keywords:** Stereotactic body radiation therapy, Fractionated radiation therapy, Primary liver cancer, Portal vein tumor thrombus, Clinical outcomes

## Abstract

**Background:**

To compare the clinical outcomes of stereotactic body radiation therapy (SBRT) and fractionated radiation therapy (FRT) for primary liver cancer with portal vein tumor thrombus (PVTT).

**Methods:**

This retrospective study included 36 patients who underwent SBRT and 36 patients who underwent FRT from August 2016 to June 2018. Patients were evaluated for short-term efficacy, long-term efficacy, AEs, and quality of life before and after treatment.

**Results:**

With a median follow-up of 28.8 months (26–36 months), 27 patients survived in the SBRT group while 19 patients survived in the FRT group. The survival rate in the SBRT group was statistically higher than that of the FRT group after 6 months (80.56% vs. 58.33%; *P* = 0.041), 12 months (77.78% vs. 55.56%; *P* = 0.046) and 24 months 75.00% vs. 52.78%; *P* = 0.049). The median whole survival time of the SBRT group was 13.3 months (95% CI 12.83–13.97), which was statistically longer than 9.8 months in the FRT group (95% CI 8.83–10.97, *P* < 0.05) based on the Kaplan–Meier method. The SBRT group had better survival quality and fewer adverse events than the FRT group.

**Conclusion:**

SBRT had better clinical outcomes than FRT for primary liver cancer with PVTT.

## Background

Primary liver cancer (PLC) is one of the most common malignant tumors clinically. More than 500,000 new cases are reported all over the world each year, making it the third most common malignant cancer [1]. China is a high incidence area of PLC, and the mortality rate of PLC is the second highest among all cancer types in China [2]. Due to atypical manifestations and an aggressive course, most PLC patients were diagnosed at the mid or advanced stage and had tumor thrombus in the main trunk or branch of the portal vein at the time of diagnosis. It has been reported that 40–90% of PLC patients have portal vein tumor thrombus (PVTT) in China [3] while 62.2–90.2% in other countries [4]. The combination of PLC and PVTT can lead to high mortality. PLC is unresectable in most patients and has poor prognosis. Besides, PLC with PVTT can induce cancer cells to metastasize into the liver, contributing to esophageal variceal bleeding and portal hypertension, so that increasing the difficulty of treatment and improving the recurrence ratet [5]. Therefore, there is an urgent need for effective radiation therapy for PLC with PVTT in the clinic.

Stereotactic body radiation therapy (SBRT) is a promising radiotherapy technology which has been widely reported in the treatment of malignant tumors [6]. The efficacy of SBRT and fractionated radiation therapy (FRT) in PLC has also been reported and both modalities showed favorable outcomes [7]. However, only few studies have been done on comparison of SBRT and FRT for PLC with PVTT. And the outcomes were not consistent with each other.

This retrospective study compared the clinical outcomes of SBRT and FRT with short-term efficacy, long-term efficacy, adverse reactions (AEs), and quality of life before and after treatment as the primary outcomes. Based on these comparisons, we aimed to determine effective radiation therapy for treating PLC with PVTT.

## Methods

This retrospective study reviewed the records of PLC patients with PVTT who received SBRT or FRT from August 2016 to June 2018 at The First Hospital of Hebei Medical University. The inclusion criteria were as follows: (1) the patients’ symptoms, imaging examination, histopathological examination, puncture cytology, and laboratory study met the diagnosis criteria of PLC with PVTT. Meanwhile, their diagnosis also met the criteria in the Diagnosis and Treatment of Primary Liver Cancer (2011, China) [8]. (2) The expected survival time was more than 6 months. (3) Normal coagulation function. (4) No contraindication for radiotherapy and chemotherapy. (5) Normal cardiac and renal function. (6) Good compliance with physician orders. The exclusion criteria were as follows: (1) patients with an infection or other malignant tumors. (2) Congenital malformation with hepatocellular carcinoma. (3) The tumor volume was more than 70% of the normal liver. (4) Metastases to other parts of the body. (5) Poor tolerance of radiation therapy. (6) Massive ascites. (7) Received prior radiotherapy.

According to these criteria, 72 patients were included in this series, 36 patients were included in the SBRT group, and 36 patients were included in the FRT group. Data regarding age, sex, follow-up duration, alpha-fetoprotein, Child–Pugh grade, the number, location and stage of the tumor, location of tumor thrombus, and stage of tumor thrombus were collected. Informed consent was obtained from all study subjects.

This study was approved by the Clinical Academic Committee of The First Hospital of Hebei Medical University and was conducted in compliance with the Helsinki Declaration.

### SBRT group

An OUR-QGD gamma-ray stereotactic body radiotherapy system was used in this study [9]. All patients were immobilized using a stereotactic body frame with a vacuum pillow to create reproducible immobilization. Then, the target areas were continuously scanned with a 3–5 mm CT-slide thickness. The obtained image data and related data were input into the planning system to construct a three-dimensional structure. Plan target volume (PTV) and clinical target volume (CTV) were delineated. According to the patient's condition, tumor location, CTV, and treatment purpose, the radiotherapy plan was customized, and dose distribution was adjusted. Thereafter, the patient was positioned and treated. In this group, the median CTV volume was 346.3 cm^3^ (range: 26–1012 cm^3^). The dose-volume histogram was used for quantitative evaluation. The median isodose curve was 55.6% (range: 50–70%). The total median dose of peripheral irradiation of PTV was 34.733 Gy (range: 3200–4800 cGy). The median fraction dose was 4.356 Gy (range: 3.800–5.200 cGy). The dose of the spinal cord and duodenum adjacent to the target area was 5–20% and 5–30%, respectively. Each patient received five sessions of treatment over 5–7 weeks.

### FRT group

This group of patients received conventional FRT. Briefly, the GE Lightspeed VCT treatment system was used in this group (Eclipse10.0, American). Patients were placed in the supine position. Then, the target areas were continuously scanned with a 3–5 mm CT-slide thickness. In addition, CT images were processed by the VARIAN Eclipse TPS planning system (VARIAN, USA). Targets were delineated following the same procedure as for the SBRT group. In this group, a single radiation dose of 2 Gy was delivered 5 times a week for 5–7 weeks. Hepatoprotective drugs were given before and during the treatment.

### Assessment of outcomes and follow-up

Both groups were evaluated for short-term efficacy, long-term efficacy, AEs, and quality of life before and after treatment as the primary outcomes.

The definition of short-term efficacy followed the RECISTI.1 criterion [10]. Short-term efficacy was defined at four levels: complete response (CR), partial response (PR), stable disease (SD) and progressive disease (PD). Each level was defined as follows: PD, the tumor volume increased by 25%, or new lesions appeared in the liver; CR, disappearance of all target lesions completely; PR, the maximum diameter of all tumors was reduced or necrotic > 50%; SD, the maximum diameter of all tumors was reduced or necrotic < 50%, or the tumor volume increase less than 25%. Response rate was the ratio of was the sum of CR and PR to whole patients. Disease control rate was the ratio of the sum of CR, PR, and SD to whole patients. The observation was started from the beginning of radiotherapy to the death or the last time follow-up.

Survival quality was evaluated using the European Organisation for Research and Treatment of Cancer Quality of Life Questionnaire Core 30 (EORTC QLQ-C30) [11]. This questionnaire included 30 items. Except items 29 and 30, each item had a score of 1–4. A higher grade represented better survival quality. Items 29 and 30 had a score of 1–7. Toxicity was evaluated using Adiation Therapy Oncology Group (RTOG) [12]. The incidence of nausea, vomiting, fatigue, myelosuppression, liver pain, and other AEs was recorded and statistically analyzed. Besides, whole survival time was recorded. The whole survival time was analyzed by the Kaplan–Meier method. All patients were followed up by telephone once a week and once a month in outpatient service for 2 years.

### Statistical analysis

All data were analyzed statistically using SPSS (version 21.0; IBM, Chicago, IL). Student’s t-test was performed to compare continuous variables. Chi-square test was performed to compare all categorical data. The survival curve was drawn by the Kaplan–Meier method. Statistical significance was set at *P* < 0.05.

## Results

The series included a total of 72 patients; the SBRT group and the FRT group each had 36 patients. Patient data, including age, sex, follow-up duration, alpha-fetoprotein, Child–Pugh grade, the number, location and stage of the tumor, location of tumor thrombus, and stage of tumor thrombus side, smoking, and follow-up time, are summarized in Table 1. There was no significant difference between the two groups in all the parameters.

**Table 1:** Comparison of clinicopathologic characteristics between SBRT group and FRT group

	SBRT (36)	FRT (36)	t/χ^2^	*P*
Gender (n)			0.058	0.81
Male	21	22		
Female	15	14		
Age	43.83 ± 6.21	43.67 ± 6.45	0.107	0.915
Alpha fetoprotein (µg/L)			0.056	0.813
≥ 400	16	17		
< 400	20	19		
Child–Pugh grade			0.296	0.586
A	26	28		
B	10	8		
Tumor number			0.056	0.813
≤ 3	19	20		
> 3	17	16		
Location (n)			0.230	0.891
Left lobe	7	6		
Right lobe	19	21		
Both sides	10	9		
Stage (n)			0.239	0.887
IIa	20	22		
IIb	10	9		
III	6	5		
Location of tumor thrombus (n)			0.397	0.982
Left branch	5	4		
Right branch	4	5		
Main portal vein + left branch	3	4		
Main portal vein + right branch	8	8		
Main portal vein + left and right branch	16	15		
Stage of tumor thrombus (n)			1.368	0.713
I	3	6		
II	11	11		
III	15	14		
IV	7	5		

### Comparison of short-term efficacy between the SBRT group and the FRT group

According to the analysis, the SBRT group had statistically better short-term efficacy than the FRT group. The response rate and disease control rate in the SBRT group were both higher than those in the FRT group (58.33% vs. 33.33%, *P* = 0.033; 88.89% vs. 69.44%, *P* = 0.042) (Table 2).

**Table 2:** Comparison of short-term efficacy between SBRT group and FRT group

Group	SBRT (n = 36)	FRT (n = 36)	χ^2^	*P*
CR (n)	5	2		
PR (n)	16	10		
SD (n)	12	12		
PD (n)	3	12		
Response rate (%)	58.33 (21/36)	33.33 (12/36)	4.531	0.033*
Disease control rate (%)	91.67 (33/36)	66.67 (24/36)	6.821	0.009*

*CR* complete response, *PR* partial response, *SD* stable disease, *PD* progressive disease. Response rate was the ratio of was the sum of CR and PR to whole patients. Disease control rate was the ratio of the sum of CR, PR, and SD to whole patients

*Have statistical difference

### Comparison of long-term efficacy between SBRT group and FRT group

The follow-up of the two groups ended on August 30, 2019, and the median follow-up duration was 28.8 months (range: 26–36 months). No patients were lost to follow up in both groups. Twenty-seven patients survived in the SBRT group, and 19 patients survived in the FRT group. The 6, 12, and 24-month survival rates in the SBRT group were statistically higher than those in the FRT group (Table 3).

**Table 3:** Comparison of survival rate between SBRT group and FRT group

Group (n)	6 months	12 months	24 months
SBRT (36)	80.56% (29/36)	77.78% (28/36)	75.00% (27/36)
FRT (36)	58.33% (21/36)	55.56% (20/36)	52.78% (19/36)
χ2	4.189	4.111	3.583
P	0.041	0.046	0.049

**Fig. 1:**
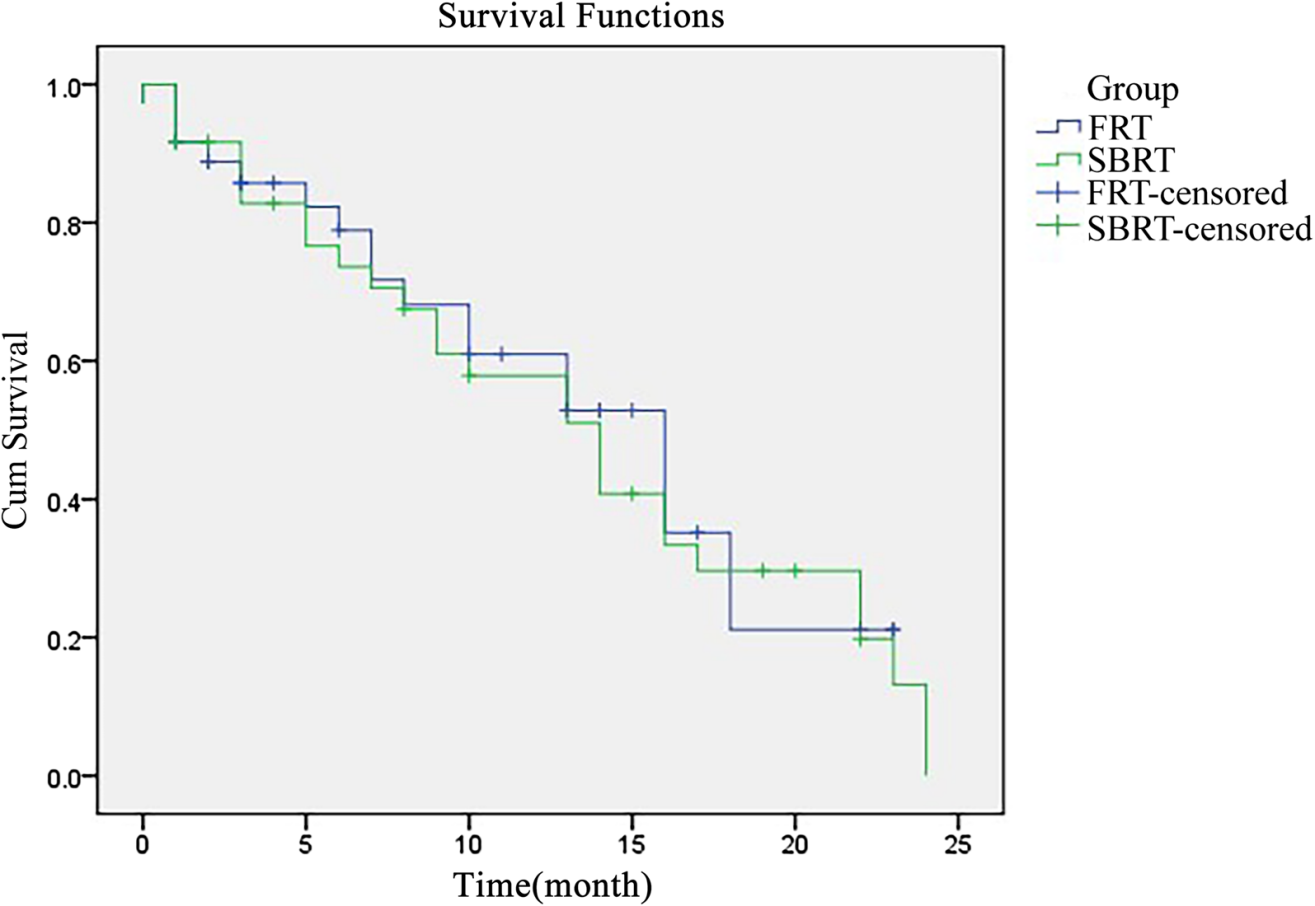
Kaplan–Meier curve of survival for both groups. SBRT, stereotactic body radiation therapy. *FRT* fractionated radiation therapy

The median whole survival time of the SBRT group was 13.3 months (95% CI 12.83–13.97), which was statistically longer than 9.8 months in the FRT group (95% CI 8.83–10.97, *P* < 0.05) (Fig. 1).

The quality of life of both groups showed no statistical difference before radiation therapy. However, both groups had better survival quality after receiving different treatments. In addition, the survival quality of the SBRT group was statistically better than that of the FRT group after therapy (Table 4).

**Table 4:** Comparison of survival quality score of EORTC QLQ-C30 between SBRT group and FRT group

Group (n)	Before treatment	After treatment	t	*P*
SBRT (36)	41.03 ± 3.13	77.73 ± 3.29	− 48.491	< 0.001
FRT (36)	39.98 ± 3.09	63.29 ± 3.01	19.43	< 0.001
t	1.432	19.430		
*P*	0.157	< 0.001		

*EORTC QLQ-C30* European Organisation for Research and Treatment of Cancer Quality of Life Questionnaire Core 30. The student’s t-test was performed to compare the data, *t* t value

During the treatment, both groups developed AEs, including nausea, vomiting, fatigue, myelosuppression, and hepatic pain. According to the RTOG grade, all symptoms were graded I–II. The incidence of liver pain, fatigue, nausea, and vomiting in the SBRT group were lower than that of the FRT group. However, the incidence of myelosuppression showed no statistical difference between the two groups. After appropriate treatment, all AEs were ameliorated. Liver failure, gastrointestinal bleeding, perforation, and other severe AEs were not reported in both groups (Table 5).

**Table 5:** Comparison of adverse reactions between SBRT group and FRT group

Group (n)	Liver function damage	Myelosuppression	Nausea and vomiting	Fatigue and dizziness
SBRT (36)	0	13.89% (5/36)	8.33% (3/36)	13.89% (5/36)
FRT (36)	11.11% (4/36)	25.00% (9/36)	27.78% (10/36)	52.78% (19/36)
χ^2^	4.235	1.419	4.603	12.25
*P*	0.039	0.234	0.032	< 0.001

## Discussion

Previous studies reported that hepatitis B virus is one of the main risk factors of liver cancer [13]. Researchers have been keeping to explore new treatment methods for the treatment of liver cancer; however, the survival time of patients has not increased significantly so far. Besides, patients with PVTT have an extremely poor prognosis, increasing the difficulty of treatment. PLC with PVTT is determined as a progressive stage according to the Barcelona Clinic Liver Cancer grade [14]. At present, sorafenib is the primary option for this disease, but it takes effect slowly and can not effectively relieve cancer cell metastasis to the liver induced by PVTT. Moreover, the therapeutic effect has certain limitations [15]. Kim et al. [16] reported that the median duration of the effects of sorafenib alone in the treatment of PLC with PVTT was less than 5 months. Therefore, it deserves to be explored whether radiotherapy combined with conventional treatment for PLC with PVTT could effectively control cancer cells, reduce the size of the lesions, maintain the normal flow of the portal vein, improve liver function and decrease the metastasis rate.

In recent years, the continuous progress of RT technology has surely significantly improved the efficiency of its application to liver cancer patients. Some studies reported that radiotherapy can effectively control tumor progression, improve long-term survival, and have a low incidence of AEs [17]. Kwon et al. [18] reported that the 1-year and 3-year disease-free survival rates were 72% and 67.5%, respectively, after radiotherapy. Andolino et al. [19] proved that the 2-year local control rate was 90%, the disease-free survival rate was 48%, and the 2-year overall survival rate was 67% for radiotherapy. The above studies confirmed the important value of radiotherapy in the treatment of liver cancer. SBRT can accurately deliver high-doses towards the tumor target area, whilst the dose outside the target’s border decreases sharply, allowing for a very low radiation exposure of the surrounding normal liver. The application of SBRT in patients with advanced liver cancer has been gradually reported by several studies. Chan used SBRT for advanced liver cancer. The outcomes showed that the 1 survival rates was 62% [20]. Similar outcomes with fewer AEs were also reported in other studies, indicating the good prospect of this method in clinical application [21, 22]. In our study, the response rate and disease control rate in the SBRT group were both statistically better than those of the FRT group. Meanwhile, the 6, 12 and 24-month survival rate, and median survival time of the SBRT group were also higher than those of the FRT group. These results were similar to those of previous studies. It indicated that SBRT yielded better clinical outcomes than FRT for PLC with PVTT. This could be explained as follows. Firstly, SBRT could reduce the pressure of the portal vein, therefore, reduce the risk of refractory ascites and esophageal variceal bleeding. Secondly, SBRT could improve the resection rate of lesions and reduce intrahepatic metastasis caused by tumor thrombus. Thirdly, it may reduce tumor load and create conditions for transcatheter arterial chemoembolization. Moreover, this method may be beneficial to portal vein blood flow and improve liver function.

In clinical practice, it is necessary to pay attention to the influence of psychological and social factors on diseases [23]. How to effectively improve the quality of life of cancer patients and prolong their life span has become a common concern in society. Therefore, this study also focused on analyzing the survival quality of PLC patients with PVTT. The results indicated that both groups had improved quality after treatment. Besides, the SBRT group had better survival quality than the FRT group. All AEs were grade I–II according to the RTOG grade. No patients had severe AEs, and the moderate symptom was relieved after appropriate treatment. The better clinical outcomes of SBRT were expected to effectively alleviate the suffering of patients.

However, this study has some limitations. Firstly, we did not collect and analyze the data of distant metastasis in PLC patients with PVTT. Secondly, the sample size was small and the duration of follow-up was brief, and a multi-center and randomized controlled trial is necessary for future work. Besides, the application of SBRT for PLC patients with PVTT, needs further research and confirmation on how to better control the liver damage and how to tailor the appropriate dose escalation depending on the size of the tumor volume.

## Conclusion

SBRT for PLC patients with PVTT has a certain curative effect and could improve the survival rate and median life cycle of patients, improving the quality of life with fewer AEs.

## Data Availability

The datasets analyzed during the current study are available from the corresponding author on reasonable request.
